# Plantar pressure is changed to increase post-impact ball speed during longline forehand and backhand groundstroke in elite female tennis players

**DOI:** 10.3389/fspor.2023.1165628

**Published:** 2023-05-18

**Authors:** Johanna Lambrich, Thomas Muehlbauer

**Affiliations:** Division of Movement and Training Sciences/Biomechanics of Sport, University of Duisburg-Essen, Essen, Germany

**Keywords:** racket sport, lower extremity, pressure-detecting insoles, plantar loading, force, biomechanics

## Abstract

**Introduction:**

Achieving high ball speed during the execution of groundstrokes represents a performance-relevant factor in tennis. However, it is unclear how plantar pressure data undergo change during the execution of groundstrokes by tennis players to achieve high postimpact ball speed. Thus, the objective of the present study is to determine how tennis players change the plantar pressure in each foot when they execute longline forehand and backhand groundstrokes in order to increase postimpact ball speed.

**Methods:**

Seventeen healthy nationally ranked female tennis players (mean age: 21.7 ± 7.7 years) participated in this study. The players performed longline forehand and backhand groundstrokes (topspin) at four postimpact ball speed levels, i.e., at 80 km/h, 90 km/h, 100 km/h, and *v*_max_. Plantar pressure was measured in each foot [i.e., dominant (equals the stroke arm) and non-dominant] using flexible instrumented insoles.

**Results:**

Irrespective of the stroke technique, the repeated measures ANOVA procedure showed significant ball speed × foot dominance interactions. For the forehand stroke, post hoc analyses revealed significantly increased (dominant foot) and decreased (non-dominant foot) pressure values when the postimpact ball speed increased from 100 km/h to *v*_max_. For the backhand stroke, the post hoc analyses yielded significantly decreased (dominant and non-dominant foot) plantar pressure values when the postimpact ball speed increased from 100 km/h to *v*_max_. There were no further significant differences between the other ball speed levels.

**Discussion:**

The significantly varying plantar pressure changes depending on the stroke technique and foot dominance to increase postimpact ball speed suggest that specific physical exercises related to the foot (dominant vs. non-dominant foot) and groundstroke (forehand vs. backhand) seem to be necessary for plantar pressure optimization.

## Introduction

In tennis, high stroke speed is an important performance-related factor, both during serves and during groundstrokes (1). In this regard, Ulbricht et al. (2) reported a higher serve speed in successful tennis players compared with less successful ones. In addition, Landlinger et al. (3) showed faster groundstrokes (i.e., forehand/backhand) in successful players than in less successful ones. In terms of groundstroke kinematics, it has been shown that more skilled players apply a higher trunk rotation during the execution of forehand (4, 5) and backhand strokes (6, 7), which results in a higher horizontal shoulder and racket speed. Moreover, Lambrich and Muehlbauer (8) found higher plantar pressure values in the forehand and backhand of advanced players compared with intermediate and recreational players. With respect to groundstrokes, the question arises as to how players change their stroke execution in order to achieve a higher ball speed. Therefore, in this study, we are interested more specifically in analyzing the changes in the movement pattern if the goal is to execute groundstrokes with increased postimpact ball speed.

To date, relatively few studies have investigated this question. For example, Seeley et al. (9) used three-dimensional high-speed video recordings to examine the kinematic changes between different postimpact ball speeds (fast: 153.7 ± 13.7 km/h; medium: 115.6 ± 10.4 km/h; slow: 77.0 ± 7.2 km/h) for the longline forehand stroke in 12 highly skilled male tennis players. They found that angles and angular velocities for several joints (i.e., ankle, knee, hip, trunk, elbow, wrist) increased significantly as the postimpact ball speed increased. Further, Shimokawa et al. (10) investigated the changes in the ground reaction force between different postimpact ball speeds (fast: 100%; medium: 90%; slow: 80%) for the cross-court forehand stroke in nine senior male and female tennis players using a force plate. Among other findings, the authors observed an increase in the peak vertical force of both feet as the postimpact ball speed increased. In addition, Rota et al. (11) found differences in the timing and level of muscle activation in the trunk and upper limbs for the forehand, with an increase ranging from 60% to 100% of individual maximum speed. Higher muscle activation was found for the external oblique, latissimus dorsi, middle deltoid, biceps brachii, and triceps brachii muscles. Although the previously reported studies have enhanced the knowledge on kinematic, kinetic, and electromyography alterations that tennis players make to increase postimpact ball speed when they execute a forehand groundstroke, studies on how players make changes in plantar pressure in each foot (i.e., dominant vs. non-dominant) to increase postimpact ball speed when executing both forehand and backhand groundstrokes are lacking.

Therefore, the aim of this study is to investigate how healthy tennis players make alterations to plantar pressure in each foot (i.e., dominant vs. non-dominant) to increase postimpact ball speed when performing longline forehand and backhand groundstrokes. We hypothesize that plantar pressure data will undergo alterations in both feet.

## Methods

### Participants

Seventeen healthy female tennis players competing in regular national tournaments participated in the study. The characteristics of the participants are presented in Table 1. Thirteen subjects were right-handed and four were left-handed, where the stroke arm corresponds to the dominant leg. All players played a one-handed forehand stroke and a two-handed backhand stroke. The inclusion criteria were female players with a national ranking of 500 or above at the time of testing. Players were excluded if they reported an illness or an actual or recent injury that was judged to potentially have an influence on stroke performance. Participants' written informed consent was obtained prior to the start of the study. The study was conducted in accordance with the Declaration of Helsinki and the human ethics committee at the University of Duisburg-Essen, Faculty of Educational Sciences, which approved the study protocol.

**Table 1:** Characteristics of the study participants (*N *= 17).

Characteristic	Value
Age (years)	21.7 ± 7.7
Body height (cm)	172.5 ± 6.4
Body mass (kg)	63.6 ± 7.8
Training experience (years)	15.3 ± 7.3
Tennis training volume (hours/week)	4.1 ± 1.8
Athletic training volume (hours/week)	4.0 ± 2.6

Data represent means ± standard deviations.

### Testing procedures

**Figure 1:**
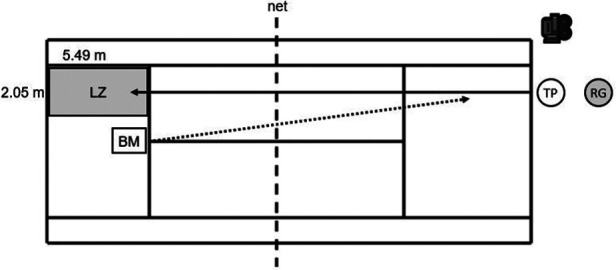
Experimental setup for a right-handed player performing longline forehand groundstrokes (topspin) using four postimpact ball speed levels (i.e., 80 km/h, 90 km/h, 100 km/h, *v*_max_). BM, ball machine; LZ, landing zone; RG, radar gun; TP, tennis player.

All measurements were performed on an indoor hardcourt (Figure 1). The players completed a familiarization phase of five minutes consisting of longline forehand and backhand groundstrokes. The feed (speed: 40 km/h, feed: 15 balls/min) was performed using a ball machine (Slinger Bag, Slinger, Windsor Mill, MD, USA). Afterward, the players were familiarized with the instrumented pressure-detecting insoles, followed by the execution of longline forehand and backhand groundstrokes (topspin) using four postimpact ball speed levels: (a) 80 km/h, (b) 90 km/h, (c) 100 km/h, and (d) *v*_max_ (feed: 15 balls/min) in a standardized order. The execution of the forehand and backhand strokes was done randomly between the players, i.e., one player executed the forehand first, followed by the backhand and then the next player in the reverse order. For conditions (a) to (c), a tolerance range of ±2 km/h was defined. The postimpact ball speed was measured to the nearest 0.16 km/h using a “Stalker Pro” radar gun (Applied Concepts Inc., Richardson, TX, USA) that was placed directly behind the players. The players were free to decide their stance (i.e., open, closed, square), and for each player, new balls were used. For each stroke, players received verbal feedback on the achieved postimpact ball speed in order to ensure that they repeatedly reached the respective predefined speed level. For each speed level, a valid trial incorporated ten successful strokes per technique in a predetermined 2.05 m × 5.49 m landing zone (Figure 1). A 60-s and a 120-s rest period was provided between speed levels and stroke techniques, respectively.

### Assessment and analysis of plantar pressure data

Flexible instrumented insoles (GP MobilData WiFi, GeBioM mbH, Münster, Germany) were used to record plantar pressure distribution at a sampling frequency of 200 Hz. These reusable insoles were placed in individual tennis shoes above the sole of the shoes. The participants used a pair of insoles according to their shoe size (e.g., a pair of insoles with a length of 250 mm corresponding to a shoe size of EU 39–40). The obtained data were recorded on a laptop via a wireless signal. Synchronously to the pressure data, the movement of each player was filmed using a video camera (iPad, Apple Inc., Cupertino, CA, USA) to determine the starting point and the impact of a stroke for subsequent analyses (Figure 1). Specifically, the plantar pressure data were normalized and interpolated from 0% to 100% of the stroke movement. A total of 201 data points were used for the interpolation. Analysis of the plantar pressure data for the whole foot of the dominant (equals the stroke arm) and the non-dominant foot was performed using Matlab software version R2022b (The MathWorks Inc., Natick, MA, USA), and it included the calculation of the normalized (to the players' body mass) maximal force (N/kg), mean force (N/kg), and force–time integral (N m s/kg).

### Statistical analysis

All statistical analyses were performed using JASP version 0.16.4.0 (Amsterdam, The Netherlands). Descriptive data are reported as group mean values and standard deviations. For all analyses, assumptions of normality (Shapiro–Wilk test) and homogeneity of variance/sphericity (Mauchly test) were confirmed prior to the application of inference statistics. Specifically, a 4 (ball speeds of 80 km/h, 90 km/h, 100 km/h, and *v*_max_) × 2 (foot dominance: dominant, non-dominant) repeated measures ANOVA was conducted for the forehand and the backhand strokes, separately. If a high ball speed occurred on account of foot dominance interaction, Bonferroni-adjusted post hoc analyses were performed. Further, the effect size (*η*_p_^2^) was calculated and reported as small (.02 ≤ *η*_p_^2^ ≤ .12), medium (.13 ≤ *η*_p_^2^ ≤ .25), or large (*η*_p_^2^ ≥ .26). The significance level was set at *p* < .05.

## Results

Descriptive statistics of the plantar pressure data by postimpact ball speeds (i.e., 80 km/h, 90 km/h, 100 km/h, and *v*_max_) and foot dominance (i.e., dominant vs. non-dominant) during the execution of longline forehand and backhand groundstrokes (topspin) are illustrated in Table 2. Maximal postimpact ball speeds amounted to 132.3 ± 8.9 km/h (range: 117–153 km/h) and 120.1 ± 7.1 km/h (range: 111–137 km/h) for the forehand groundstroke and backhand groundstroke, respectively.

**Table 2:** Descriptive statistics of the plantar pressure data by postimpact ball speed level (i.e., 80 km/h, 90 km/h, and 100 km/h, *v*_max_) and foot dominance (i.e., dominant vs. non-dominant) during longline forehand and backhand groundstrokes (topspin).

Outcome	80 km/h		90 km/h		100 km/h		*v*_max_	
	D	ND	D	ND	D	ND	D	ND
Forehand stroke								
Maximal force (N/kg)	1.08 ± 0.31	0.91 ± 0.18	1.09 ± 0.22	0.90 ± 0.22	1.12 ± 0.23	0.86 ± 0.25	1.25 ± 0.31	0.70 ± 0.26
Mean force (N/kg)	0.56 ± 0.18	0.30 ± 0.07	0.54 ± 0.17	0.29 ± 0.07	0.56 ± 0.18	0.28 ± 0.09	0.62 ± 0.17	0.19 ± 0.08
Force–time integral (N m s/kg)	56.21 ± 17.99	29.56 ± 7.41	54.32 ± 17.33	28.88 ± 7.16	56.52 ± 18.35	27.60 ± 8.67	62.42 ± 17.54	19.26 ± 7.90
Backhand stroke								
Maximal force (N/kg)	1.12 ± 0.15	1.16 ± 0.16	1.14 ± 0.21	1.24 ± 0.17	1.14 ± 0.21	1.22 ± 0.17	1.02 ± 0.21	1.19 ± 0.17
Mean force (N/kg)	0.35 ± 0.05	0.57 ± 0.09	0.33 ± 0.06	0.60 ± 0.09	0.33 ± 0.07	0.60 ± 0.10	0.27 ± 0.06	0.56 ± 0.08
Force–time integral (N m s/kg)	34.50 ± 5.34	57.42 ± 9.28	32.54 ± 6.30	60.07 ± 8.63	32.54 ± 6.61	60.78 ± 9.56	27.06 ± 5.91	56.16 ± 7.81

Values are expressed as mean ± standard deviation. D, dominant foot; ND, non-dominant foot.

### Forehand stroke

**Figure 2:**
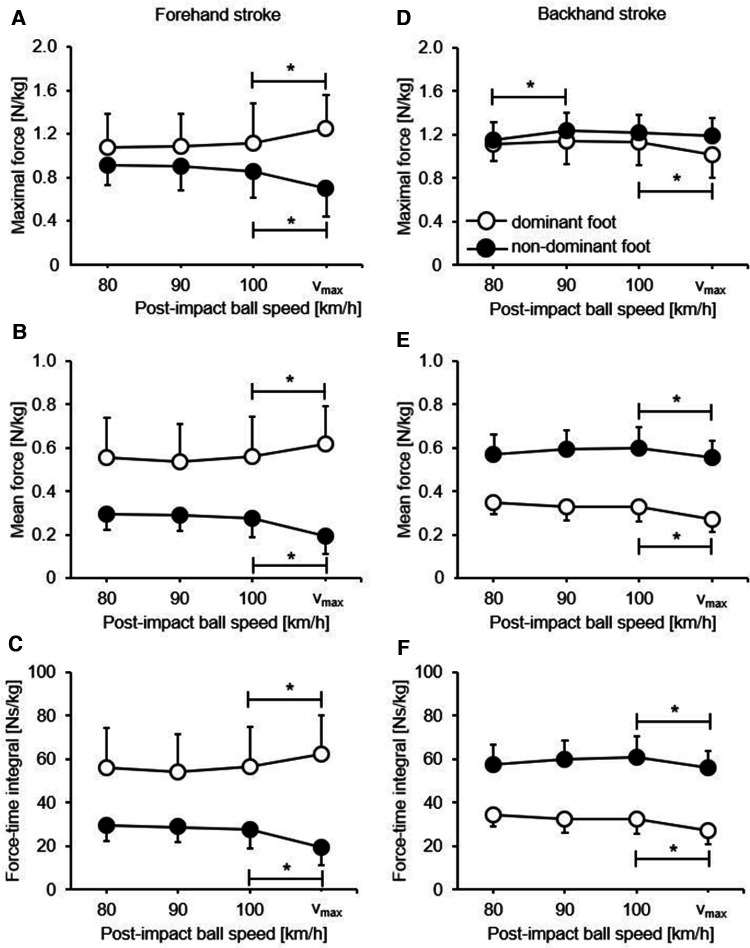
Plantar pressure values (mean and standard deviation) per postimpact ball speed level for the dominant (white) vs. non-dominant (black) foot during the execution of longline forehand (**A–C**) and backhand (**D–F**) groundstrokes (topspin). *Represents a significant difference between speed levels.

Irrespective of the outcome measure, the ANOVA revealed significant main effects of foot dominance (*p *< .001–.004, *η*_p_^2^ = .30–.67), indicating higher values for the dominant foot compared with that for the non-dominant foot, but not for ball speed (Table 3). Further, there were significant ball speed × foot dominance interaction effects (all *p *< .001, *η*_p_^2^ = .04–.08). Post hoc analyses revealed that plantar pressure values significantly increased in the dominant foot (maximal force: *p *= .012; mean force: *p *= .010; force–time integral: *p *= .010) but significantly decreased in the non-dominant foot (maximal force: *p *= .002; mean force: *p *< .001; force–time integral: *p *< .001) when players increased their postimpact ball speed from 100 km/h to *v*_max_ (Figures 2A–C). There were no further significant differences between the other ball speed levels.

**Table 3:** Inference statistics for the main and interaction effects.

Outcome	Main effect: BS	Main effect: FD	Interaction effect: BS × FD
Forehand stroke			
Maximal force (N/kg)	.943 (.01)	.004 (.30)	<.001 (.08)
Mean force (N/kg)	.389 (.01)	<.001 (.67)	<.001 (.04)
Force–time integral (N m s/kg)	.332 (.01)	<.001 (.66)	<.001 (.04)
Backhand stroke			
Maximal force (N/kg)	.002 (.11)	<.001 (.23)	.008 (.05)
Mean force (N/kg)	<.001 (.02)	<.001 (.90)	.002 (.01)
Force–time integral (N m s/kg)	<.001 (.02)	<.001 (.89)	.001 (.08)

Values are expressed as *p*-value (*η_p_*^2^-value). BS, ball speed; FD, foot dominance.

### Backhand stroke

The ANOVA showed significant main effects of ball speed (*p *< .001–.002, *η*_p_^2^ = .02–.11) and foot dominance (all *p *< .001, *η*_p_^2^ = .23–.90) as well as a significant interaction (*p *= .001–.002, *η*_p_^2^ = .01–.08) between the two irrespective of outcome measure (Table 3). Values for the dominant foot were lower than those for the non-dominant foot. For the dominant foot, post hoc analyses showed that plantar pressure values (maximal force: *p *= .001; mean force: *p *< .001; force–time integral: *p *< .001) significantly decreased to increase the post-impact ball speed from 100 km/h to *v*_max_ (Figures 2D–F). There were no further significant differences between the other ball speed levels. For the non-dominant foot, mean force (*p *= .002) and force–time integral (*p *= .002) but not maximal force significantly decreased when the aim was to increase the post-impact ball speed from 100 km/h to *v*_max_ (Figures 2D–F). However, there was a significant increase in maximal force (*p *< .001) as the backhand stroke was executed to hit the ball with 80 km/h rather than 90 km/h.

## Discussion

We aimed to examine the changes made by healthy nationally ranked female tennis players to plantar pressure in each foot when they executed longline forehand and backhand groundstrokes to increase postimpact ball speeds (i.e., 80 km/h, 90 km/h, 100 km/h, and *v*_max_). In accordance with our assumption, we detected that plantar pressure data were significantly altered in both feet to increase postimpact ball speed during the execution of longline forehand and backhand groundstrokes. However, plantar pressure changes differed by foot dominance and the stroke technique. On the one hand, values were higher and increased in the dominant foot (equals the stroke arm) but were lower and decreased in the non-dominant foot during the execution of the forehand stroke to increase the postimpact ball speed from 100 km/h to *v*_max_. On the other hand, values decreased in the dominant and non-dominant foot during the performance of the backhand stroke to increase the postimpact ball speed from 100 km/h to *v*_max_ and were lower in the dominant foot than in the non-dominant foot. There were no further significant differences between the other ball speed levels.

With regard to the forehand stroke, Shimokawa et al. (10) also observed an increase in kinetic parameters (e.g., peak vertical force) as the post-impact ball speed increased from slow to medium and to fast, yet the authors did not distinguish between the dominant and the non-dominant foot but analyzed both feet together. A possible reason for the players significantly increasing the plantar pressure in their dominant foot in order to increase the postimpact ball speed from 100 km/h to *v*_max_ could be attributed to the fact that due to the higher force in the dominant leg, a higher rotation in the pelvis and trunk is achieved, resulting in a higher stroke velocity (9, 12, 13). In this context, Nesbit et al. (14) found that the range of motion of the dominant knee also contributes significantly to hip and trunk rotation. In contrast, the observed significant decrease in plantar pressure in the non-dominant foot as the forehand stroke is hit with increased post-impact ball speed could most likely be explained by the fact that the dominant leg has primarily a stabilizing function (15). Moreover, according to Shimokawa et al. (10), the horizontal force becomes greater as the stroke velocity increases. From a practical perspective, the increase in plantar pressure in the dominant foot suggests that a combination of bilateral and unilateral exercises such as leg press, dumbbell lunge, and squat should be used in conditioning programs to improve the vertical force component (16). In contrast, the decrease in plantar pressure in the non-dominant foot indicates a declining importance of the vertical force component. Therefore, exercises to increase other force components such as horizontal force (e.g., jump-landing tasks such as single/triple hops for distance, side jumps, and sprints) and to improve its stabilizing function (e.g., high skipping with a single leg halt, medicine ball chest passes while standing on one leg, and lateral raises while standing on one leg on an unstable surface) seem to be more appropriate (16–18).

With respect to the backhand stroke, the decrease in plantar pressure in both feet as the postimpact ball speed increased from 100 km/h to *v*_max_ could be explained by the fact that the players shifted their body mass more forward in the direction of the stroke. As a result, the horizontal force increased and the vertical force decreased. In fact, a closer look at the video recordings (see Methods section) indicated that the increase in the postimpact ball speed from 80 to 90 km/h was only due to an increased arm movement, but from 90 to 100 km/h, it was due to an increased arm and leg movement, and from 100 km/h to *v*_max_, it was due to an increased whole-body movement in the direction of the stroke. It can be assumed that, since the use of the lower extremity in the two-handed backhand is comparable to the forehand (19), an increased stroke velocity is also the result of a higher horizontal force (10). In terms of practical implications for coaches and players, it can be deduced that conditioning programs aiming to improve horizontal force should be devised.

During the execution of the forehand stroke, plantar pressure values for the dominant foot (equals the stroke arm) were greater than those for the non-dominant foot (Figures 2A–C), but this was the opposite for the backhand stroke (Figures 2D–F). This indicates a varying role of foot dominance depending on the stroke technique. For the forehand and in accordance with Chen et al. (15), larger pressure values in the dominant vs. non-dominant foot seem to be favorable, as the dominant foot is responsible for force production and the non-dominant one is meant for body stabilization. With reference to Elliott et al. (20) and Knudson (21), the two-handed backhand shows an analogy to a non-dominant forehand regarding the use of the lower limbs. Specifically, the non-dominant foot now takes the force-generating function, while the dominant foot stabilizes the balance of the body. These results are consistent with those of Akutagawa and Kojima (19), who found a higher joint movement in the non-dominant leg compared with the dominant leg during the performance of the two-handed backhand stroke. This explains the observed larger plantar pressure values in the non-dominant vs. dominant foot during the backhand stroke execution.

This study has some strengths and limitations. In terms of strengths, for the first time, changes in plantar pressure data were measured for the longline forehand and the backhand groundstrokes with increased postimpact ball speed. Moreover, these measurements were not performed in a laboratory setting but on the tennis court and thus under natural field-based conditions. Furthermore, the investigations were carried out with a homogeneous group of individuals (i.e., healthy nationally ranked female tennis players). With regard to the limitations, the use of pressure-detecting insoles had the disadvantage that only an indirect but not direct determination of force data (e.g., by means of a plate) was possible. Further, only kinetic but not kinematic data were collected. Thus, no spatiotemporal description of the groundstroke movements could be made, but only the vertical forces that cause the strokes were analyzed. In addition, only female tennis players were studied, limiting the transferability of our findings to male players, who should be investigated in future studies. Lastly, only 17 players were studied. Although this sample size was larger than that in previous studies (*n *= 12 in the study by Seeley and colleagues and *n *= 9 in the study by Shimokawa and colleagues), future investigations should use larger samples to strengthen the findings of this study.

## Conclusion

This study investigated how plantar pressure data changed as the longline forehand and backhand groundstrokes (topspin) were executed with increased postimpact ball speeds (i.e., 80 km/h, 90 km/h, 100 km/h, and *v*_max_). During the execution of the forehand stroke, pressure data increased in the dominant foot and decreased in the non-dominant foot when the goal was to increase the postimpact ball speed from 100 km/h to *v*_max_. While performing the backhand stroke, pressure data decreased on both feet (i.e., dominant and non-dominant foot) in order to achieve an increase in the postimpact ball speed from 100 km/h to *v*_max_. The varying changes in the plantar pressure data as a function of stroke technique and foot dominance suggest the need for specifically tailored physical exercises for the dominant vs. non-dominant foot and for the longline forehand vs. backhand stroke in female tennis players.

## Data Availability

The original contributions presented in the study are included in the article, further inquiries can be directed to the corresponding author.
